# Urachal Cyst Causing Small Bowel Obstruction in an Adult with a Virgin Abdomen

**DOI:** 10.1155/2016/3247087

**Published:** 2016-11-09

**Authors:** Michael P. O'Leary, Zane W. Ashman, David S. Plurad, Dennis Y. Kim

**Affiliations:** Department of Surgery, Harbor-UCLA Medical Center, Torrance, CA, USA

## Abstract

*Introduction*. A patent urachus is a rare congenital or acquired pathology, which can lead to complications later in life. We describe a case of urachal cystitis as the etiology of small bowel obstruction in an adult without prior intra-abdominal surgery.* Case Report*. A 64-year-old male presented to the acute care surgery team with a 5-day history of right lower quadrant abdominal pain, distention, nausea, and vomiting. He had a two-month history of urinary retention and his past medical history was significant for benign prostate hyperplasia. On exam, he had evidence of small bowel obstruction. Computed tomography revealed high-grade small bowel obstruction secondary to presumed ruptured appendicitis. In the operating room, an infected urachal cyst was identified with adhesions to the proximal ileum. After lysis of adhesions and resection of the cyst, the patient was subsequently discharged without further issues.* Conclusion*. Although rare, urachal pathology should be considered in the differential diagnosis when evaluating a patient with small bowel obstruction without prior intraabdominal surgery, hernia, or malignancy.

## 1. Introduction

Surgical evaluation for small bowel obstruction by an acute care surgical team is common. Etiologies include, but are not limited to, adhesions secondary to prior intra-abdominal operations, hernias, and malignancy. However, urachal pathology can also lead to inflammation, adhesions, and mechanical small bowel obstruction.

Numerous studies have described the management of urachal pathology [1–5]. However, urachal cystitis as an etiology of mechanical small bowel obstruction is rare. The purpose of this descriptive report is to recognize urachal pathology as a differential diagnosis in adult patients presenting with small bowel obstructions without prior intra-abdominal operations.

## 2. Case Presentation

A 64-year-old male presented to the emergency department with a 5-day history of right lower quadrant abdominal pain and distention. He had nausea, vomiting, obstipation, and anorexia. He denied fevers and chills. His past medical history was significant for benign prostate hyperplasia and a two-month history of urinary retention for which he had been followed up by the urology service. He denied previous abdominal surgeries. Examination revealed a distended tympanic abdomen with bilateral lower abdominal quadrant tenderness without rebound or guarding. Laboratory values revealed a white blood cell count of 15,100 cells/mcL with 89% neutrophilia. A urinalysis contained 182 white blood cells, 3+ leukocytes, and 4+ bacteria with a subsequent urine culture growing* Escherichia coli*.

**Figure 1:**
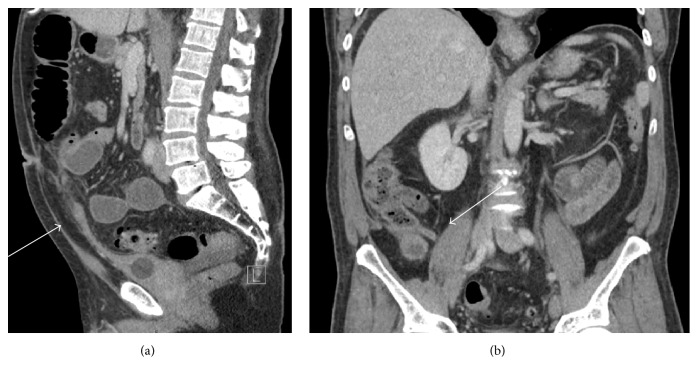
CT scan demonstrating a Foley catheter in the decompressed bladder with (a) demonstration of a urachal cyst connection from the anterior reflection of the bladder to the umbilicus with dilated small bowel loops and adjacent fat stranding and (b) a normal appearing posterior appendix off the cecum.

A computed tomography (CT) scan was performed which demonstrated fat stranding in the right inferior abdominal cavity with an adjacent fluid collection, distended small bowel loops with fecalization, and a clear transition point at the proximal ileum. The preliminary radiologic interpretation was that of an intra-abdominal abscess secondary to a ruptured appendicitis with associated high-grade small bowel obstruction. Following the insertion of a nasogastric tube and the administration of antibiotics, the patient was taken to the operating room. Prior to incision, the CT scan was reexamined and a fluid collection with surrounding fat stranding was noted extending from the bladder to the umbilicus with a transition point at the phlegmon (Figure 1(a)). Moreover, a normal appendix was visualized (Figure 1(b)).

**Figure 2:**
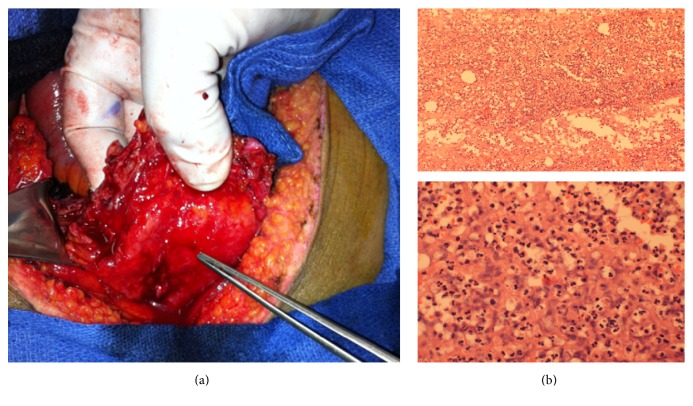
Intraoperative and pathology specimens showing anatomical and microscopic inflammatory findings consistent with urachal cyst as the etiology for small bowel obstruction. (a) Urachal cyst in the inferior right intraperitoneal cavity as the cause of small bowel obstruction. (b) Microscopic inflammation with severe neutrophilia.

Upon entry into the peritoneal cavity, a 4 × 5 centimeter cystic mass was encountered inferiorly and to the right of the umbilicus which was adherent and in continuity with the bladder (Figure 2(a)). The proximal ileum was adherent to this process and was identified as the transition point. The small bowel in its entirety was examined and there was no evidence of any further pathology. The urology service was consulted intraoperatively and an urachal cystotomy revealed a small amount of purulent fluid. Digital exploration of the mass revealed no obvious connection with the bladder. Indigo carmine was injected through the Foley catheter, which did not enter the cystic lesion. The specimen was excised. Pathology revealed fibroadipose tissue with severe and chronic inflammation consistent with urachal cystitis (Figure 2(b)). Cultures were consistent with his urinary tract infection, namely, light growth of* Escherichia coli*. The patient was discharged on postoperative day 9 following an uncomplicated course in hospital.

## 3. Discussion

Urachal pathology has been described in the literature for a century. The categorical description with regard to epidemiology and diagnostic modalities has only recently been described. In the developing fetus, the bladder descends into the pelvis and the apical aspect of the urachus transforms into a fibromuscular strand between the transversalis fascia anteriorly and peritoneum posteriorly called the space of Retzius. Normal vestigial structures are between 3 and 10 cm in length in adults and symptomatic pathology involves dilatation of the urachus [1].

**Figure 3:**
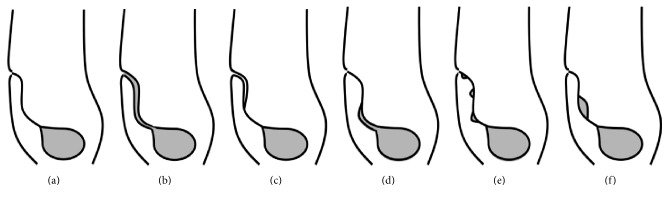
Congenital and acquired urachal abnormalities. (a) Normal urachus; (b) congenital patent urachus; (c) acquired umbilical urachal sinus; (d) acquired vesicourachal diverticulum; (e) acquired alternating urachal sinuses; (f) acquired urachal sinus.

Two main urachal pathologies are described, congenital and acquired. A congenital patent urachus is typically identified in childhood by urine draining via the umbilicus. The acquired pathology has been further categorized into four types: (1) an umbilical urachal sinus, whereby the tract is dilated at the umbilicus and opens directly to the atmosphere, (2) a vesicourachal diverticulum, which compromises any short segment dilatation of the proximal portion of the urachus, (3) alternating urachal sinuses with fibromuscular tissue interposed, and (4) a urachal cyst involving a noncontiguous short segment dilatation of the urachus (Figure 3) [1]. The largest review of urachal cyst pathology reviewed three hundred and fifteen cases with nearly half being attributed to the congenital patent urachus. The acquired anomaly compromised 52.4%. Of the acquired type, the urachal cyst comprised 58.8% and was most prone to present as an infected process [2]. Acquired urachal pathology can be difficult to diagnose clinically as the associated inflammation can mimic many disease processes and mandate a broad differential diagnosis.

Urachal pathology may have similar presentations as incarcerated umbilical hernia, acute appendicitis, Meckel's diverticulitis, or peritonitis secondary to bowel perforation. Initial diagnosis of urachal pathology is challenging. For example, a recent report described a young male without medical problems or surgical history who presented with periumbilical pain after heavy lifting. A preliminary diagnosis of umbilical hernia was made. Surgical evaluation confirmed the presence of urachal cyst [3]. Intraperitoneal disease processes can be challenging to distinguish as well. In our case report, acute appendicitis was initially suspected as the etiology of his high-grade obstruction given the brevity of symptoms, localization of pain, and inflammation on preliminary CT read. However, careful analysis of the CT scan findings revealed the correct diagnosis. In patients with an acute surgical abdomen from intraperitoneal rupture of the cystic contents, the diagnosis is even more difficult preoperatively [4].

Infection of urachal cysts is rare beyond the 5th decade of life. Most frequently, the infection occurs in infancy. Patients may present with pain, periumbilical erythema, and leukocytosis. The space of Retzius, which contains the urachal pathology, is extraperitoneal and management options include incision and drainage of the preperitoneal infection with packing and subsequent excision of the cyst [5]. Given that the lesions are extraperitoneal, associated inflammation extending intraperitoneally should be recognized as advanced disease. In patients who present with small bowel obstruction secondary to infected urachal cysts, this may represent a chronic process leading to adhesions. Important clinical indicators of early infection must not be overlooked so as to avoid intraperitoneal progression of the disease process. We feel that surgical management for patients with high-grade small bowel obstruction secondary to adhesions from recurrent urachal infections should address (1) the small bowel obstruction with lysis of adhesions and resection of ischemic small bowel and (2) excision of the cyst and urachal remnant with bladder repair as needed. Excision should be performed to prevent recurrent disease and to rule out malignancy.

Urachal remnants progressing to malignancy are rare, compromising less than 0.01 percent of cancers. Case reports have described the progression of disease into adenocarcinoma, which occurs in the 5th to 7th decade of life, and sarcoma, which occurs in adults prior to the 5th decade [6, 7]. Adenocarcinomas of the urachal ligament have poor response to chemoradiation. Surgical resection for patients with resectable disease has improved disease-free survival; however, 5-year survival is of 40 percent. Improved long-term survival can be obtained with negative resection margins and absence of nodal involvement [8]. Hence, in the setting of known malignancy associated with urachal pathology, margin negative, en bloc resection should be attempted.

Diagnostic modalities are limited in adults. CT has been used to identify adjacent pathology and the extent of the disease but CT has limitations. Namely, fat stranding can mask the true underlying pathology and not allow identification of the space of Retzius. Urachal cystitis may also appear as an intraabdominal phlegmon, as with our case [1]. The utility of ultrasonography is also limited in diagnosing urachal pathology but may be underutilized as a diagnostic modality in the emergency department. Benefits of real time ultrasound include a faster and more sensitive diagnosis from improved visualization of the cystic structure. Also the cystic pathology is away from intestinal gas and is located in the anterior abdominal wall limiting bowel gas interference. Lesions as small as 2 centimeters have been identified on ultrasound. In infants, ultrasound has up to 100% sensitivity when identifying a patent urachus, 100% sensitivity for urachal sinus, and 82% sensitivity with urachal cysts [9, 10]. Therefore, children should be evaluated using predominantly sonography. In adult patients who are stable to proceed to CT, the initial use of ultrasonography may be used to establish the diagnosis. However, CT should also be performed with specific attention to the sagittal views for visualization of the Retzius space [11]. Moreover, a CT scan appropriately stages a patient in the rare case of malignant transformation of the urachal remnant.

Although uncommon, an infected urachal cyst should be considered in the differential diagnosis when evaluating an adult with small bowel obstruction without previous abdominal surgeries and a clear transition point near the anteroinferior peritoneal wall. In children, ultrasound should be used to diagnose urachal pathology. Adults benefit from both an ultrasound and a CT scan to rule out malignant transformation. In the case of urachal cystitis in an adult causing small bowel obstruction, the surgical management should include the standard management of small bowel obstruction with excision of the urachal remnant to exclude malignancy.

## Additional Points

Urachal cystitis can cause small bowel obstruction in a virgin abdomen and should be considered in the differential diagnosis.

## References

[1] Berman S. M., Tolia B. M., Laor E., Reid R. E., Schweizerhof S. P., Freed S. Z. (1988). Urachal remnants in adults. *Urology*.

[2] Blichert-Toft M., Nielsen O. V. (1971). Diseases of the urachus simulating intra-abdominal disorders. *The American Journal of Surgery*.

[3] Ash A., Gujral R., Raio C. (2012). Infected urachal cyst initially misdiagnosed as an incarcerated umbilical hernia. *Journal of Emergency Medicine*.

[4] Ohgaki M., Higuchi A., Chou H. (2003). Acute peritonitis caused by intraperitoneal rupture of an infected urachal cyst: report of a case. *Surgery Today*.

[5] Lees V. C., Doyle P. T. (1991). Urachal cyst presenting with abscess formation. *Journal of the Royal Society of Medicine*.

[6] Mattelaer P., Wolff J. M., Jung P., IJzerman W., Jakse G. (1997). Adenocarcinoma of the urachus: 3 case reports and a review of the literature. *Acta Urologica Belgica*.

[7] Tsiouris A., Ahmed H. U., Kumar N., Kaisary A. V. (2007). Urachal tumour: clinical and radiological features of a poorly understood carcinoma. *Annals of the Royal College of Surgeons of England*.

[8] Siefker-Radtke A. O., Gee J., Shen Y. (2003). Multimodality management of urachal carcinoma: the M. D. Anderson Cancer Center experience. *The Journal of Urology*.

[9] Morin M. E., Tan A., Baker D. A., Sue H. K. (1979). Urachal cyst in the adult: ultrasound diagnosis. *American Journal of Roentgenology*.

[10] Yiee J. H., Garcia N., Baker L. A., Barber R., Snodgrass W. T., Wilcox D. T. (2007). A diagnostic algorithm for urachal anomalies. *Journal of Pediatric Urology*.

[11] Yu J.-S., Kim K.-W., Lee H.-J., Lee Y.-J., Yoon C.-S., Kim M.-J. (2001). Urachal remnant diseases: spectrum of CT and US findings. *RadioGraphics*.

